# The prevalence and clinical characteristics of pertussis-associated pneumonia among infants in Botswana

**DOI:** 10.1186/s12887-019-1820-0

**Published:** 2019-11-16

**Authors:** Bahaa Abu-Raya, David M. Goldfarb, Marek Smieja, Kathy Luinstra, Melissa Richard-Greenblatt, Andrew P. Steenhoff, Kristen A. Feemster, Tonya Arscott-Mills, Coleen K. Cunningham, Samir S. Shah, Mohamed Zaakir Patel, Matthew S. Kelly, Manish Sadarangani

**Affiliations:** 1 Vaccine Evaluation Center, BC Children’s Hospital Research Institute, University of British Columbia, 950 West 28th Avenue, Vancouver, BC V5Z 4H4 Canada; 2 Division of Infectious Diseases, Department of Pediatrics, University of British Columbia, Vancouver, BC Canada; 3 Botswana-University of Pennsylvania Partnership, Gaborone, Botswana; 4 Department of Pathology and Laboratory Medicine, BC Children’s Hospital, Vancouver, British Columbia Canada; 5 Department of Pathology and Molecular Medicine, McMaster University, Hamilton, Ontario Canada; 6 Division of Infectious Diseases, Children’s Hospital of Philadelphia, Philadelphia, PA USA; 7 Global Health Center, Children’s Hospital of Philadelphia, Philadelphia, PA USA; 8 Perelman School of Medicine, University of Pennsylvania, Philadelphia, PA USA; 9 Division of Pediatric Infectious Diseases, Duke University Medical Center, Durham, NC USA; 10 Divisions of Hospital Medicine and Infectious Diseases, Cincinnati Children’s Hospital Medical Center, Cincinnati, OH USA; 11 Department of Paediatrics and Adolescent Health, University of Botswana School of Medicine, Gaborone, Botswana; 12 Division of Disease Control, Philadelphia Department of Public Health, Philadelphia, PA USA

**Keywords:** *Bordetella pertussis*, HIV-exposed uninfected, Pneumonia

## Abstract

**Background:**

There are scant data on the prevalence and clinical course of pertussis disease among infants with pneumonia in low- and middle-income countries. While pertussis vaccination coverage is high (≥90%) among infants in Botswana, human immunodeficiency virus (HIV) infection affects nearly one-third of pregnancies.

We aimed to evaluate the prevalence and clinical course of pertussis disease in a cohort of HIV-unexposed uninfected (HUU), HIV-exposed uninfected (HEU), and HIV-infected infants with pneumonia in Botswana.

**Methods:**

We recruited children 1–23 months of age with clinical pneumonia at a tertiary care hospital in Gaborone, Botswana between April 2012 and June 2016. We obtained nasopharyngeal swab specimens at enrollment and tested these samples using a previously validated in-house real-time PCR assay that detects a unique sequence of the porin gene of *Bordetella pertussis*.

**Results:**

*B. pertussis* was identified in 1/248 (0.4%) HUU, 3/110 (2.7%) HEU, and 0/33 (0.0%) HIV-infected children. All pertussis-associated pneumonia cases occurred in infants 1–5 months of age (prevalence, 1.0% [1/103] in HUU and 4.8% [3/62] in HEU infants). No HEU infants with pertussis-associated pneumonia were taking cotrimoxazole prophylaxis at the time of hospital presentation. One HUU infant with pertussis-associated pneumonia required intensive care unit admission for mechanical ventilation, but there were no deaths.

**Conclusions:**

The prevalence of pertussis was low among infants and young children with pneumonia in Botswana. Although vaccination against pertussis in pregnancy is designed to prevent classical pertussis disease, reduction of pertussis-associated pneumonia might be an important additional benefit.

## Background

Pertussis is a respiratory illness most commonly caused by *Bordetella pertussis*. The disease is classically divided into catarrhal, paroxysmal, and convalescent stages. The paroxysmal stage is classical of pertussis and manifests as intense bouts of coughing which may be interrupted by a high-pitched “whoop” as the child rapidly inhales. However, paroxysmal cough may be absent in young infants, and pertussis disease in infancy can be complicated by apnea, convulsions, and death [1].

The burden of pertussis disease in low- and middle-income countries (LMICs) is difficult to estimate and poorly recorded. This may be due to the limited availability of diagnostic assays for *B. pertussis* detection and under recognition of pertussis infection that is not associated with classical clinical features [2]. Several factors that might affect the risk and severity of pertussis disease among infants merit special consideration in LMICs. Of particular concern is maternal human immunodeficiency virus (HIV) infection, which is estimated to affect nearly one-third of pregnancies in Botswana and several other countries in sub-Saharan Africa [3]. HIV-exposed uninfected (HEU) infants are at higher risk of severe infections [4] and have lower anti-pertussis antibodies at birth than the infants of HIV-negative mothers (HIV-unexposed uninfected; HUU) [5].

There are scant data on the prevalence and clinical course of pertussis among infants with pneumonia in LMICs because most studies selectively enrolled infants with clinical symptoms classically associated with pertussis (e.g., whooping cough). As infants and young children with pertussis disease often do not manifest these classical symptoms, restricting recruitment to patients with these symptoms has likely led to underestimation of pertussis disease prevalence. Furthermore, immunization against pertussis in pregnancy is being implemented in an increasing number of countries, with the primary goal of preventing classical pertussis disease among young infants [6]. However, an additional benefit of maternal pertussis immunization programs may be the reduction of pneumonia caused by pertussis. Thus, data on the prevalence and clinical course of pertussis-associated pneumonia are important in settings where immunization against pertussis during pregnancy is being considered.

The aim of this study was to evaluate the prevalence and clinical course of pertussis disease in a cohort of HUU, HEU, and HIV-infected infants and young children with pneumonia in Botswana.

## Methods

### Study population

The study population was previously described in detail [7]. Briefly, we recruited children 1–23 months of age presenting with pneumonia at Princess Marina Hospital in Gaborone, Botswana between April 2012 and June 2016. We defined pneumonia per World Health Organization (WHO) clinical criteria as “cough or difficulty in breathing with lower chest wall indrawing” [7]. We excluded children with chronic medical conditions predisposing to pneumonia (except HIV infection), hospitalization within the previous 14 days, a diagnosis of asthma, or wheezing with resolution of chest wall indrawing after ≤2 treatments with a bronchodilator. Whole-cell pertussis (wP) vaccine is used for primary and booster immunization of infants and children in Botswana, with the primary series being administered at 2, 3, and 4 months of age. Women were not routinely immunized for pertussis during pregnancy in Botswana throughout the study period.

### Laboratory methods

We obtained nasopharyngeal swab specimens from all children at enrollment using flocked swabs and universal transport media (Copan Italia, Brescia, Italy). Specimens were stored at − 80 °C, shipped on dry ice at 6-month intervals to the Regional Virology Laboratory (St. Joseph’s Healthcare, Hamilton, ON, Canada), and tested for respiratory viruses using PCR [7]. In the analyses presented herein, we used a previously validated highly sensitive and specific in-house real-time PCR assay that detects a unique sequence of the porin gene of *B. pertussis* [8].

## Results

*B. pertussis* was identified in 4/396 (1.0%) children with pneumonia, including 3/110 (2.7%) HEU children, 1/248 (0.4%) HUU children, and 0/33 (0.0%) HIV-infected children. All pertussis-associated pneumonia cases were among infants 1–5 months of age (Table 1). Among infants 1–5 months of age, the prevalence of *B. pertussis* was 1.0% (1/103) in HUU infants and 4.8% (3/62) in HEU infants. No HEU infants with pertussis-associated pneumonia were taking cotrimoxazole prophylaxis at the time of hospital presentation. Among HEU infants 1–5 months of age who tested negative for *B. pertussis*, 30/62 (48%) were taking cotrimoxazole at the time of presentation. Among the 20 HIV-infected children with data on cotrimoxazole prophylaxis, 9 (45%) were taking cotrimoxazole at the time of presentation.

**Table 1:** Characteristics of infants 1–23 months of age with pertussis-associated pneumonia in Gaborone, Botswana, 2012–2016

Clinical information	Case 1	Case 2	Case 3	Case 4
Demographics				
Year admitted	2013	2014	2015	2014
Maternal age (years)	39	26	42	39
Infant age (months)	1	2	2	4
Sex	F	M	F	F
Birth weight < 2500 g	No	No	Yes	No
HIV exposure status	HUU	HEU	HEU	HEU
In utero ART exposure	None	None	HAART	HAART
Infant zidovudine	No	Yes	Yes	Yes
Nutrition and infant feeding practices				
Duration of breast feeding (months)	2	0	0	5
Severe malnutrition	No	No	No	No
Vaccination status and prophylaxis				
Pertussis-containing vaccine (DPT-HEPB-HIB) doses received	0	1	1	3
Cotrimoxazole prophylaxis	NA	No	No	No
Clinical features				
URI	Yes	Yes	Yes	Yes
Fever	No	Yes	Yes	No
Duration of cough prior to admission (days)	14	7	14	3
WBC (*10^9^ cells/L)	20.4	7	22.3	9.5
WHO severe disease^a^	No	No	Yes	No
Hypoxia on admission^c^	No	No	No	No
Hospital course and outcome				
O2 requirement (days)	11	0	4	0
CPAP	No	No	No	No
Admission to ICU	Yes	No	No	No
Intubation (days)	4	0	0	0
Treatment^d^	Third generation cephalosporin	None	Third generation cephalosporin + vancomycin	None
LOS (days)	15	2	8	2
Survival	Yes	Yes	Yes	Yes

Abbreviations: *M* Male, *F* Female, *HEU* HIV-exposed uninfected, *HUU* HIV-unexposed uninfected, *NA* Not applicable, *WHO* World Health Organization, *LOS* Length of stay, *ART* Anti-retroviral therapy, *HAART* Highly active antiretroviral therapy, *URI* Upper respiratory infection, *CPAP* Continuous positive airway pressure, *ICU* Intensive care unit, *LOS* Length of stay

^a^Pneumonia accompanied by WHO danger signs (central cyanosis, convulsions, inability to drink, or abnormal sleepiness)

^b^Weight-for-length < −3 standard deviation on standard WHO growth curves, mid-upper arm circumference < 115 mm (for children < 6 months of age), or bilateral edema of nutritional origin

^c^Hypoxia was defined as oxygen saturation < 90% on room air

^d^The decision to administer antibiotics and choice of antibiotic was made by the pediatrician

Respiratory virus co-infection was detected in two infants with pertussis-associated pneumonia, including one infant with respiratory syncytial virus and one infant with rhinovirus B. One 4-month-old infant with pertussis-associated pneumonia had completed a 3-dose series of primary immunization against pertussis. None of the infants with pertussis-associated pneumonia received antibiotics active against *B. pertussis* during the hospitalization. One HUU infant with pertussis-associated pneumonia required intensive care unit admission for mechanical ventilation, but there were no deaths.

## Discussion

*B. pertussis* was detected from only 1% of children less than 2 years of age with pneumonia in Botswana. However, among infants 1–5 months of age with pneumonia, the prevalence of *B. pertussis* was 2%, including a prevalence of approximately 5% among HEU infants in this age group. Pertussis-associated pneumonia was not associated with mortality in the few cases observed in this cohort. This study provides important data on the prevalence and clinical outcome of pertussis-associated pneumonia among infants and young children in a LMIC setting where surveillance for pertussis disease is lacking and wP vaccine is used for routine childhood immunization against pertussis.

The prevalence of *B. pertussis* among infants 1–5 months of age with pneumonia in this study is similar to the prevalence among infants 1–5 months of age (2.6%; 40/1547) and HEU infants aged 1–5 months of age (4.7%; 12/256) hospitalized with severe or very severe pneumonia in multiple African countries (The Gambia, Kenya, Mali, South Africa, Zambia) in the Pneumonia Etiology Research for Child Health (PERCH) Study [9]. Our results are also similar to a study conducted in South Africa, where the prevalence of *B. pertussis* was 2.3% (42/1839) among infants < 12 months of age (95% of whom were < 6 months of age) and 2.7% (16/599) in HIV-exposed infants hospitalized with respiratory illnesses or neonatal sepsis [10]. However, we observed a lower prevalence of pertussis than was seen in a study of South African children < 13 years of age hospitalized with lower respiratory tract infection (LRTI), in which the prevalence of *B. pertussis* was 7.0% (32/460) among all children (median age 8 months) and 10.9% (10/92) in HEU children [11].

Several factors may influence the prevalence of *B. pertussis* among infants in our cohort compared with previous studies. Vaccination coverage for three doses of pertussis vaccine is higher in Botswana than in South Africa (≥90% vs. 66%) [12], and ranges between 45 and 99% in other African countries [13]. Moreover, acellular pertussis (aP) vaccine is used in South Africa for primary immunization [9], while wP vaccine is used in Botswana and in most other African countries [13]. Children who receive wP vaccine have greater protection against pertussis disease than children who receive aP vaccine [14]. Moreover, we may have underestimated the prevalence of pertussis disease by using nasopharyngeal samples for microbiological diagnosis. In the aforementioned South African study [11], nearly half (47%) of pertussis cases were positive by PCR from induced sputum samples and negative by PCR from nasopharyngeal samples. In another study, induced sputum increased the diagnostic yield for *B. pertussis* over nasopharyngeal swabs alone by 18% among infants < 6 months of age with respiratory illnesses [15]. However, nasopharyngeal swabs are used more commonly than induced sputum for diagnostic testing in young infants, and our approach is likely to be representative of that used in clinical practice.

The prevalence of pertussis among infants 1–5 months of age was higher in HEU than HUU infants, but the small number of cases precluded meaningful statistical analysis. A difference would be theoretically plausible given previous data that HEU infants have lower anti-pertussis antibodies at birth compared with HUU infants [5]. Notably, none of the HEU infants < 6 months of age with pertussis-associated pneumonia were receiving cotrimoxazole prophylaxis at the time of presentation, but nearly half of infants who tested negative for *B. pertussis* were taking this medication.

Cotrimoxazole is a broad-spectrum antimicrobial agent used to treat a range of bacteria as well as some fungi and protozoa and is a treatment option for pertussis disease. WHO guidelines recommend use of cotrimoxazole prophylaxis for all infants of HIV-infected mothers, from 4 to 6 weeks of age until the end of HIV exposure and until HIV infection has been excluded [16]. However, these guidelines have been debated and the benefit of cotrimoxazole prophylaxis to HEU infants remains unclear [17, 18]. A recent trial did not find an overall survival benefit from cotrimoxazole prophylaxis among HEU infants in Botswana [19]. Cotrimoxazole has antimicrobial activity against a wide range of pathogens and its routine and widespread use could increase the development of bacterial resistance [20]. In addition, there is a potential risk of increasing sulfadoxine-pyrimethamine resistance in *Plasmodium falciparum* [21].

In this study, pertussis was not detected in any of the 33 HIV-infected infants with pneumonia. The transplacental transfer of anti-pertussis antibodies is reduced in HIV-infected women relative to HIV-uninfected women [22]. However, the clinical significance of this observation is unclear due to the lack of an established serum antibody level that provides clinical protection against pertussis disease. It is possible that the lower anti-pertussis antibody levels in HIV-exposed infants are still protective against pertussis disease. In addition, a proportion of HIV-infected infants (9/20 with available prophylaxis data) were on cotrimoxazole prophylaxis, which could have reduced their risk of *B. pertussis* infection*.*

Although the prevalence of pertussis among infants with pneumonia in this study is relatively low, identification of *B. pertussis* may still be of importance in young infants as pertussis disease can be complicated by apnea, convulsions, and death in this population [1]. Although not observed among the small number of infants with pertussis in this study, these complications might be evident at the population level. There are scarce and conflicting data regarding the effect of *B. pertussis* detection on the severity of LRTI among infants. The identification of *B. pertussis* among infants with LRTI was previously associated with a relatively mild clinical presentation and course compared to infants from whom *B. pertussis* was not detected [23]. However, in the PERCH Study, infants < 6 months of age with pertussis-associated pneumonia were more likely to have vomiting and high leukocyte counts (> 20,000 cells/μL) than infants with pneumonia who tested negative for pertussis [9].

Antibiotic treatment is indicated for all cases of pertussis, regardless of the clinical presentation or the severity of the illness. While treatment of pertussis early in the course of the disease is more likely to ameliorate symptoms than treatment later in the disease course [24], an additional benefit of treatment of pertussis disease is the reduction of person-to-person transmission [25–27]. Interrupting transmission is an important public health benefit because there is high prevalence of subclinical or mild infections among household contacts of pertussis cases, which may play a critical role in the maintenance of ongoing transmission of pertussis [27].

Immunization against pertussis in pregnancy has been shown to decrease the risk of pertussis infection and the severity of pertussis disease among young infants [28, 29]. An additional benefit to pertussis immunization in pregnancy may be the prevention of illnesses that are not classically associated with pertussis. Given that pertussis vaccination in pregnancy is highly effective (~ 85% efficacy) in preventing pertussis disease among young infants [29], it is also reasonable to anticipate that the majority of pertussis-associated pneumonia cases in this study could have been prevented by pertussis immunization in pregnancy.

This study has a number of strengths. *B. pertussis* was tested for in a large number of infants with pneumonia in an African country with long-standing high uptake of the wP vaccine. The study was prospective and conducted over 5 years, thus minimizing the possibility that seasonal variation and the cyclical pattern of pertussis might influence the results. This study also has several important limitations. It was conducted at a single referral center and the results may not be generalizable to non-tertiary care centers. Furthermore, patients with more severe pneumonia may have died before admission to hospital, and consequently some cases of pertussis may have been missed. The small sample size of HIV-infected infants limits our ability to draw definitive conclusions about the prevalence of *B. pertussis* among infants with pneumonia in this population. Finally, the study did not include infants < 1 month of age, an age group that is highly susceptible to severe pertussis infection.

## Conclusion

The prevalence of pertussis was low among infants and young children with pneumonia in Botswana. The limited data on the burden of pertussis in young infants in LMICs is a major obstacle to determining if vaccination against pertussis in pregnancy warrants implementation in such settings. Although vaccination against pertussis in pregnancy is designed to prevent classical pertussis disease, reduction of pertussis-associated pneumonia might be an important additional benefit.

## Data Availability

The datasets used and/or analysed during the current study are available from the corresponding author on reasonable request.

## Abbreviations

aP: Acellular pertussis
HEU: HIV-exposed uninfected
HIV: Human immunodeficiency virus
HUU: HIV-unexposed uninfected
LMICs: Low- and middle-income countries
LRTI: Lower respiratory tract infection
PCR: Polymerase chain reaction
WHO: World Health Organization
wP: Whole-cell pertussis
